# Peripheral huntingtin silencing does not ameliorate central signs of disease in the B6.*Htt^Q111/+^* mouse model of Huntington’s disease

**DOI:** 10.1371/journal.pone.0175968

**Published:** 2017-04-28

**Authors:** Sydney R. Coffey, Robert M. Bragg, Shawn Minnig, Seth A. Ament, Jeffrey P. Cantle, Anne Glickenhaus, Daniel Shelnut, José M. Carrillo, Dominic D. Shuttleworth, Julie-Anne Rodier, Kimihiro Noguchi, C. Frank Bennett, Nathan D. Price, Holly B. Kordasiewicz, Jeffrey B. Carroll

**Affiliations:** 1 Behavioral Neuroscience Program, Psychology Department, Western Washington University, Bellingham, WA, United States of America; 2 Institute for Genome Sciences and Department of Psychiatry, University of Maryland School of Medicine, Baltimore, MD, United States of America; 3 Institute for Systems Biology, Seattle, WA, United States of America; 4 Department of Mathematics, Western Washington University, Bellingham, WA, United States of America; 5 INSERM U1216, Grenoble Institute of Neuroscience, Grenoble, France. Université Grenoble Alpes, Grenoble, France; 6 Ionis Pharmaceuticals, Carlsbad, CA, United States of America; Centre de Recherche Jean-Pierre Aubert, FRANCE

## Abstract

Huntington’s disease (HD) is an autosomal dominant neurodegenerative disease whose predominant neuropathological signature is the selective loss of medium spiny neurons in the striatum. Despite this selective neuropathology, the mutant protein (huntingtin) is found in virtually every cell so far studied, and, consequently, phenotypes are observed in a wide range of organ systems both inside and outside the central nervous system. We, and others, have suggested that peripheral dysfunction could contribute to the rate of progression of striatal phenotypes of HD. To test this hypothesis, we lowered levels of huntingtin by treating mice with antisense oligonucleotides (ASOs) targeting the murine *Huntingtin* gene. To study the relationship between peripheral huntingtin levels and striatal HD phenotypes, we utilized a knock-in model of the human HD mutation (the B6.*Htt*^*Q111/+*^ mouse). We treated mice with ASOs from 2–10 months of age, a time period over which significant HD-relevant signs progressively develop in the brains of *Htt*^*Q111/+*^ mice. Peripheral treatment with ASOs led to persistent reduction of huntingtin protein in peripheral organs, including liver (64% knockdown), brown adipose (66% knockdown), and white adipose tissues (71% knockdown). This reduction was not associated with alterations in the severity of HD-relevant signs in the striatum of *Htt*^*Q111/+*^ mice at the end of the study, including transcriptional dysregulation, the accumulation of neuronal intranuclear inclusions, and behavioral changes such as subtle hypoactivity and reduced exploratory drive. These results suggest that the amount of peripheral reduction achieved in the current study does not significantly impact the progression of HD-relevant signs in the central nervous system.

## Introduction

Huntington’s disease (HD) is an autosomal dominant neurodegenerative disorder caused by a glutamine-encoding CAG expansion near the 5’ end of the *HTT* gene [1]. The symptoms of HD are progressive cognitive, affective, and motor deficits, generally beginning in mid-life and progressing inexorably to death approximately 15 years after initial symptoms present [2]. These symptoms have been linked to progressive dysfunction and cell death in corticostriatal circuits in mutation carriers [3], though widespread atrophy is also implicated to varying degrees [4]. By late stages of the disease, nearly complete loss of striatal projection cells has occurred [5].

Due to HD’s purely genetic etiology, complete penetrance, and the wider interest in the pre-symptomatic progression of neurodegenerative diseases, HD mutation carriers have been intensively studied. Several studies have characterized progressive phenotypes in asymptomatic HD mutation carriers, in some cases for longer than 10 years of continuous observation [6,7]. These observational studies reveal widespread peripheral signs associated with carrying the HD mutation, in addition to progressive neurological symptoms [8]. The appearance of peripheral phenotypes may not be surprising, given that the Huntingtin protein (HTT) and transcript (*HTT*) are widely, and consistently, expressed in every cell type so far studied [9]. Observed peripheral changes in HD mutation carriers include subtly enhanced immune activation [10], progressive reductions in hepatic mitochondrial function [11,12], and progressive loss of lower limb strength [13]. We have proposed [9] that these symptoms are worth understanding for several reasons: first–peripheral dysfunction may contribute to CNS pathology directly; second–they may directly lead to patient morbidity and/or mortality; and finally–they may uncover novel aspects of HTT function by revealing physiological pathways impacted by its mutation in other organs.

We set out to test the first of these hypotheses by peripherally silencing HTT in the B6.*Htt*^*Q111/+*^ mouse model of the HD mutation, which mimics the genetics of HD by expressing a single mutant allele from the endogenous murine Htt locus [14]. Compared to HD patients and transgenic mouse models, these mice have relatively subtle signs of disease but do present with a wide range of molecular changes, especially in the striatum, the most vulnerable region of the brain in HD [15,16]. To study the relationship between peripheral huntingtin levels and disease, we silenced huntingtin (and mutant huntingtin) using peripherally-restricted antisense oligonucleotides (ASOs). ASOs are short strands of chemically-modified deoxyribonucleotides which hybridize with target mRNA and modulate its processing in various ways, including RNaseH-mediated degradation [17]. Because they are large, charged molecules, peripherally-administered ASOs are effectively excluded from the brain by the blood-brain barrier [18]. We took advantage of this selective localization to investigate whether peripheral silencing of HTT would impact signs of the disease in the central nervous system (CNS). We were particularly interested in the impact of hepatic HTT silencing on HD symptoms because the liver is an important nexus for brain-body cross-talk. The liver synthesizes glucose (via gluconeogenesis or glycogenolysis) and ketone bodies (via ketogenesis), which serve as critical substrates for the brain between meals and while fasting [19]. The liver also regulates whole-body levels of nitrogenous waste products, including urea, a critical function for the preservation of brain health. Increased brain urea levels have been reported in human HD patients and model sheep [19,20]. Likewise, increased circulating ammonia has been reported in mouse models of disease [21]. Liver failure is generally associated with neurological sequelae, hepatic encephalopathy, which, like HD, involves dysfunction in corticostriatal circuits, increased inflammation, and excitotoxicity [22].

Given the important links between peripheral organ function and brain health, and evidence that the HD mutation is associated with alterations in whole-body physiology, we tested the relationship between peripheral huntingtin silencing and striatal signs of HD in the B6.*Htt*^*Q111/+*^ model of HD. Using systemically-delivered ASOs, we silenced hepatic HTT during a window in which a range of progressive striatal signs of disease develop. This treatment robustly reduced HTT levels in liver and adipose tissues, but did not alter striatal signs of HD, suggesting these are independent of hepatic dysfunction in this model at this age.

## Results

### HTT knockdown and body weight

**Fig 1:**
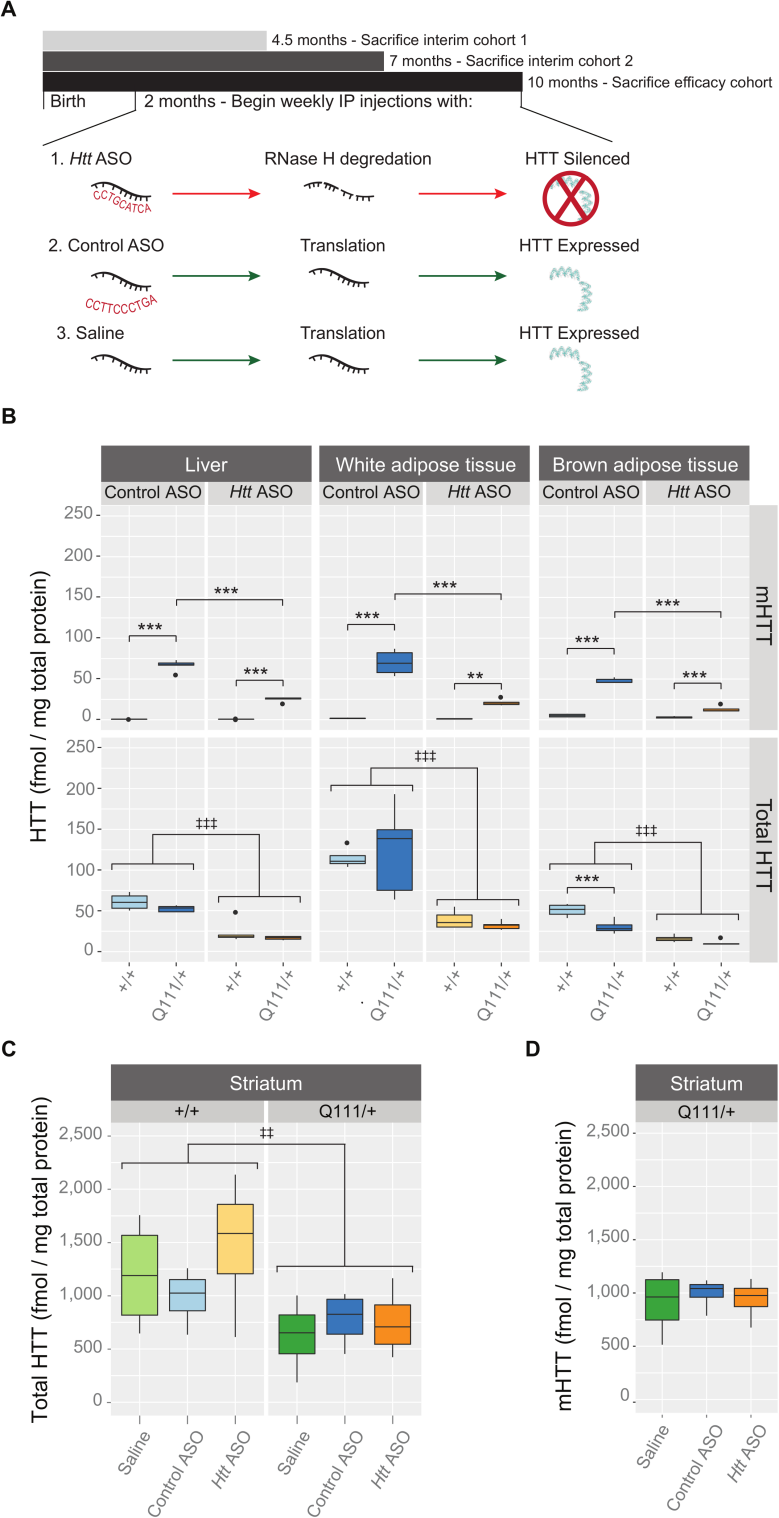
IP-delivered *Htt* ASO suppresses both wild-type and mutant huntingtin in the periphery, but not striatum. (A) Starting at 2 months of age and continuing until 10-months, *Htt*^*Q111/+*^ and *Htt*^*+/+*^ mice received weekly IP injections of *Htt* ASO, control ASO, or saline alone. In parallel to this efficacy cohort, two interim cohorts were established to verify continuous suppression of HTT by *Htt* ASO. (B) Levels of total and mutant HTT were quantified by MSD assay in three peripheral tissues: liver, white and brown adipose tissue. *Htt* ASO treatment significantly reduced HTT levels compared to control ASO treatment in all three tissues. (C-D) Treatment with *Htt* ASO did not alter striatal levels of total (B) or mutant (C) huntingtin. Mutant HTT was not detectable in the striatum of *Htt*^+/+^ mice, therefore data are only presented for *Htt*^Q111/+^ mice in (D). All data are presented as boxplots. * p ≤ 0.05, ** p ≤ 0.01, *** p ≤ 0.001: by Tukey’s HSD pairwise comparisons ‡ p ≤ 0.05, ‡‡ p ≤ 0.01, ‡‡‡ p ≤ 0.001: by factorial ANOVA. Abbreviations: white adipose tissue (WAT), brown adipose tissue (BAT).

We intraperitoneally (IP) injected a pan-*Htt*-targeted ASO (hereafter ‘*Htt* ASO’), an off-target control ASO (hereafter ‘control ASO’), or saline to suppress total HTT in *Htt*^*Q111/+*^ and *Htt*^*+/+*^ littermates from 2- to 10-months of age (Fig 1A). Peripheral HTT knockdown by *Htt* ASO treatment was confirmed in three tissues of interest—liver, perigonadal white adipose tissue, and interscapular brown adipose tissue—using mesoscale discovery (MSD) assays, which quantify total and polyglutamine expanded HTT (Fig 1B) [23]. Compared to control ASO treated mice, *Htt* ASO treated mice had significantly reduced HTT levels in all peripheral tissues examined (effect of treatment in the liver: *F*_(1, 15)_ = 79.6, *p* = 2.2 x 10^−7^, white adipose tissue: *F*_(1, 15)_ = 39.6, *p* = 1.4 x 10^−5^, and brown adipose tissue: *F*_(1, 15)_ = 89.2, *p* = 1.1 x 10^−7^). To confirm the non-allele-specificity of the chosen *Htt* ASO, mutant HTT (mHTT) levels were independently determined using an antibody pair specific for mutant huntingtin. Consistent with the identical sequence between wild-type and mutant *Htt* at the ASO target, similar mHTT suppression patterns are observed in all peripheral tissues tested (effect of treatment in the liver: *F*_(1, 15)_ = 163.2, *p* = 1.8 x 10^−9^, white adipose tissue: *F*_(1, 15)_ = 53.2, *p* = 2.7 x 10^−6^, and brown adipose tissue: *F*_(1, 15)_ = 233.3, *p* = 1.5 x 10^−10^). In contrast to its effects in peripheral tissues, *Htt* ASO had no effect on total HTT (*F*_(2, 18)_ = 0.73, *p* = 0.49) or mutant HTT (*F*_(2, 9)_ = 0.16, *p* = 0.86) levels in the striatum (Fig 1C and 1D). Among peripheral tissues examined, *Htt* ASO treatment suppressed total HTT to similar extents, ranging from 64% knockdown in the liver to 71% knockdown in white adipose tissue. These data confirm robust HTT silencing in the examined peripheral organs (liver, white adipose, and brown adipose) without reducing levels in the striatum. To verify HTT silencing throughout the trial, we examined two interim cohorts (Fig 1A) and found silencing consistent with the endpoint analysis described above (S3 Fig). As a qualitative assessment of *Htt* ASO uptake in other peripheral tissues, we stained fixed organs with an antibody reactive to the ASO backbone. We find that *Htt* ASO uptake appears most efficient in the liver, spleen, and kidney, with modest uptake in the heart and skeletal muscle (S4 Fig).

**Fig 2:**
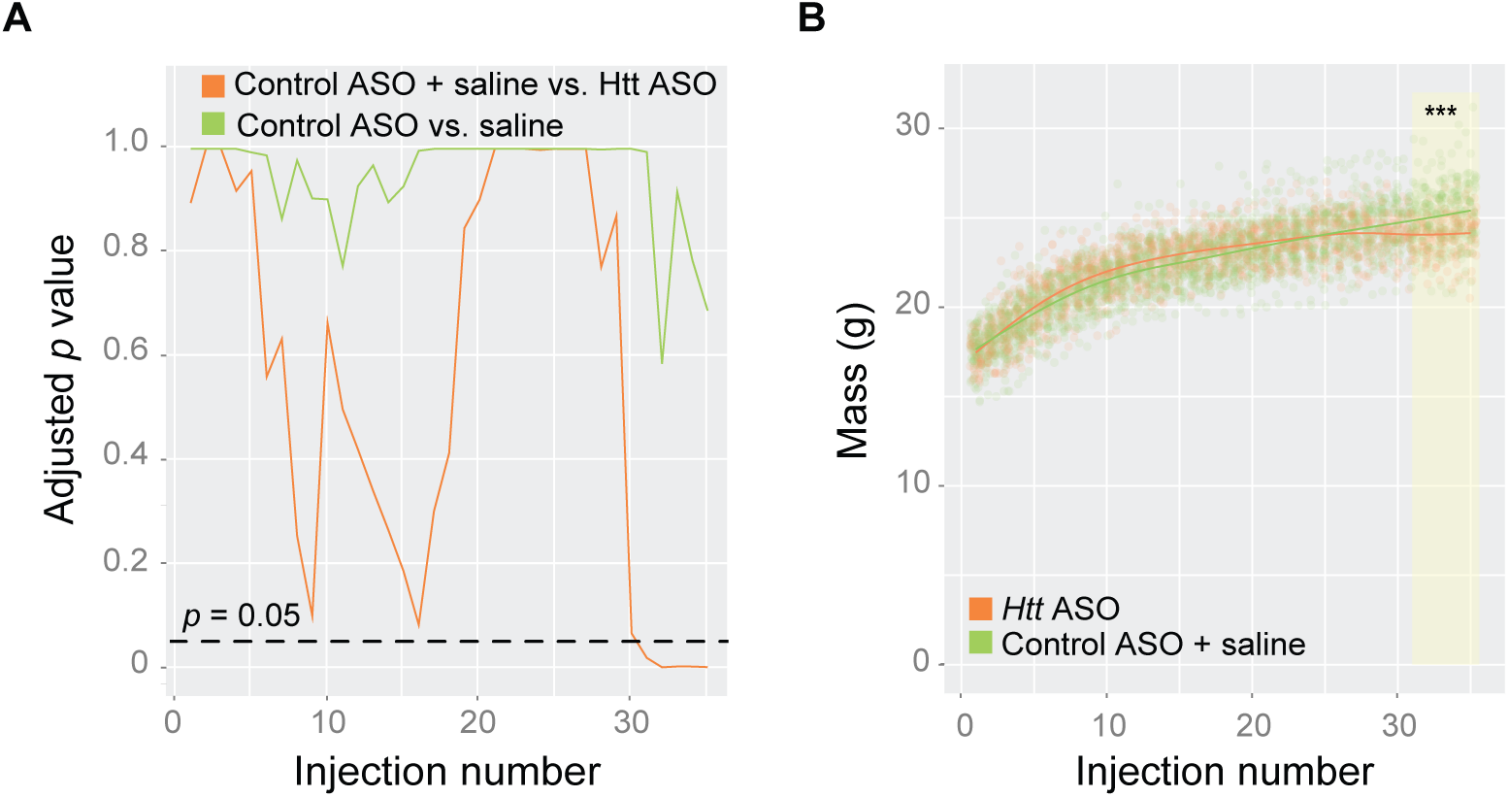
Prolonged treatment with *Htt* ASO leads to modest reduction in body weight. Body mass was recorded weekly during IP injections and examined longitudinally using minP-based parametric bootstrap multiple comparison procedure [24][25]. (A) No significant differences were observed between control ASO and saline treated mice (*p* = 0.58; green); therefore, control ASO and saline treated mice were grouped and compared to *Htt* ASO treated mice (*p* < 0.05 after 30 injections; orange) (B) Body mass was reduced by 5% in *Htt* ASO -treated mice after 30 injections compared to grouped control ASO and saline treated mice (yellow highlighted region; overall curve comparison p = 0.0001).

To quantitate differences in body mass throughout the 35 weeks of treatment, we used a minP-based parametric bootstrap multiple comparison procedure [24,25] to account for multiple measurements. Consistent with previous observations [16,26], we did not observe changes in body weight in *Htt*^*Q111/+*^ mice relative to wild-types (*P*_min_ = 0.27, *p* = 0.92). Therefore, we solely focused on the treatment effects by pooling weight observations from both the *Htt*^*Q111/+*^ and *Htt*^*+/+*^ mice. As we were interested in the mean weight difference (if any) between saline and control ASO treated mice, as well as the mean weight difference between the grouped saline and control ASO treated mice compared to *Htt* ASO treated mice, these two hypotheses were tested simultaneously. After 30 weeks of *Htt* ASO treatment, mice exhibit modestly decreased body mass (Fig 2B), weighing 5% less than grouped control and saline treated mice (Fig 2A; *P*_min_ < 0.001, *p* = 0.0001). There was no significant difference at any week between the saline and control ASO treated mice (*P*_min_ = 0.04, *p* = 0.58, Fig 2A).

### Central pathological signs

**Fig 3:**
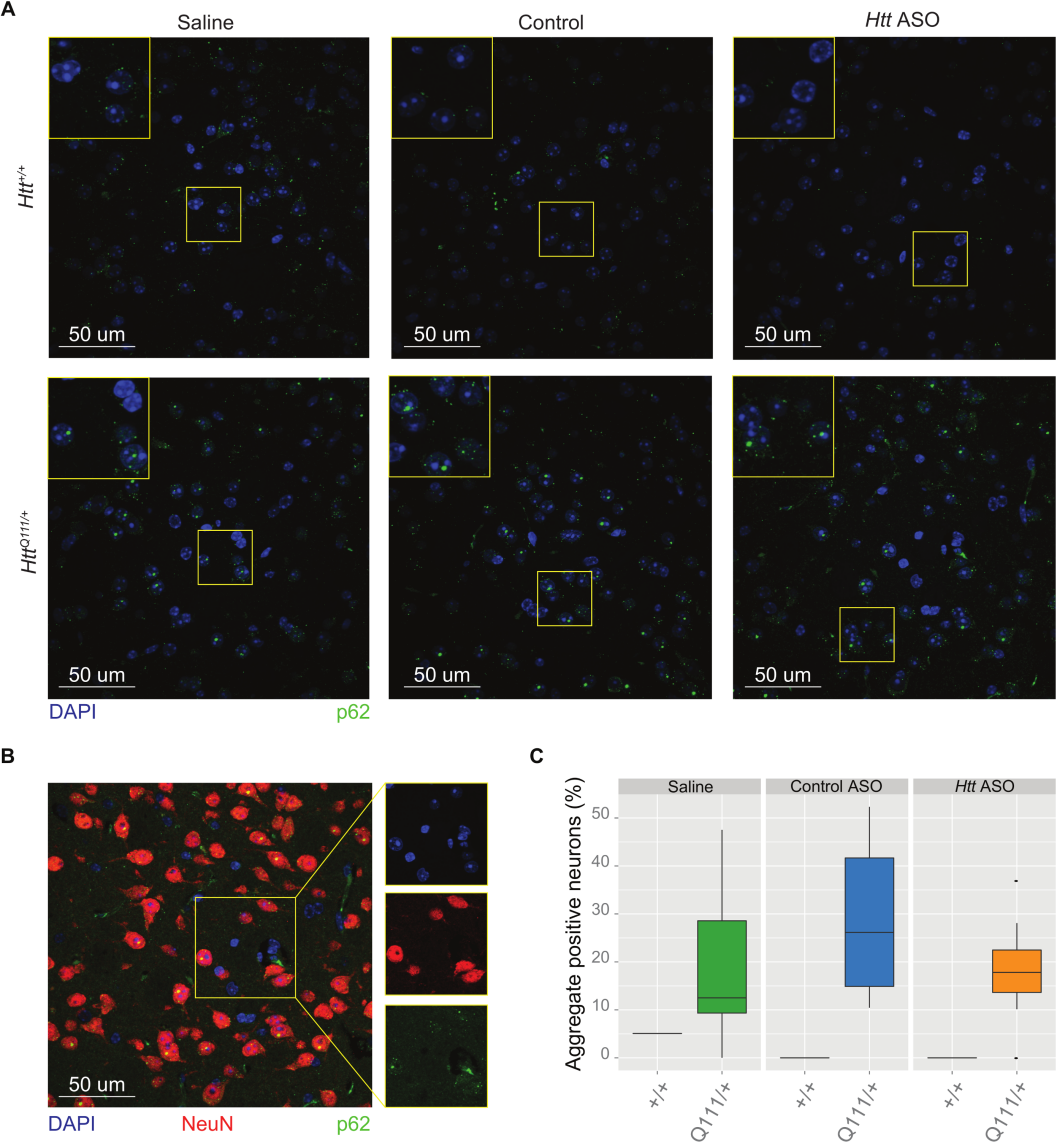
Peripheral *Htt* silencing does not prevent formation of p62-immunoreactive neuronal intranuclear inclusions in the striatum. (A) Representative images taken of the dorsolateral striatum show nuclei (DAPI, blue) and autophagy adapter p62 (green) for each genotype and treatment condition. For clarity, staining of neuronal marker NeuN was omitted in (A). However, a neuronal (NeuN) mask was used to selectively quantify p62 aggregates in neurons, therefore a representative image of an *Htt*^*Q111/+*^ mouse is shown in (B). Quantification of staining among *Htt*^*Q111/+*^ mice (C) reveals no differences in the percent of neurons with intranuclear inclusions between treatment groups.

Striatal-specific accumulation of neuronal intranuclear inclusions (NII’s) is a defining feature of the aging *Htt*^*Q111/+*^ mouse [16]. We therefore considered whether peripheral suppression of HTT affected central aggregate load by counting aggregates immunoreactive for p62/Sqstm-1, a receptor for cargos destined to be degraded by selective macroautophagy [27]. In order to restrict our analysis to neurons, we created a mask for our images by selecting only cells immunoreactive for Rbfox3 (also known as NeuN), a pan-neuronal marker protein [28]. Consistent with previous investigations [16], we observe accumulation of p62-immunoreactive NII’s in striatal neurons from *Htt*^*Q111/+*^ mice (22.5 ± 0.14% of *Htt*^*Q111/+*^ striatal neurons have NIIs (0.5–5 μm^2^ size cutoff) compared to 0 ± 0.3% of *Htt*^*+/+*^ neurons, Fig 3). Peripheral treatment of *Htt*^*Q111/+*^ mice with *Htt* ASO does not impact this phenotype (effect of treatment: *F*_(2,24)_ = 1.6, *p* = 0.2, Fig 3).

**Fig 4:**
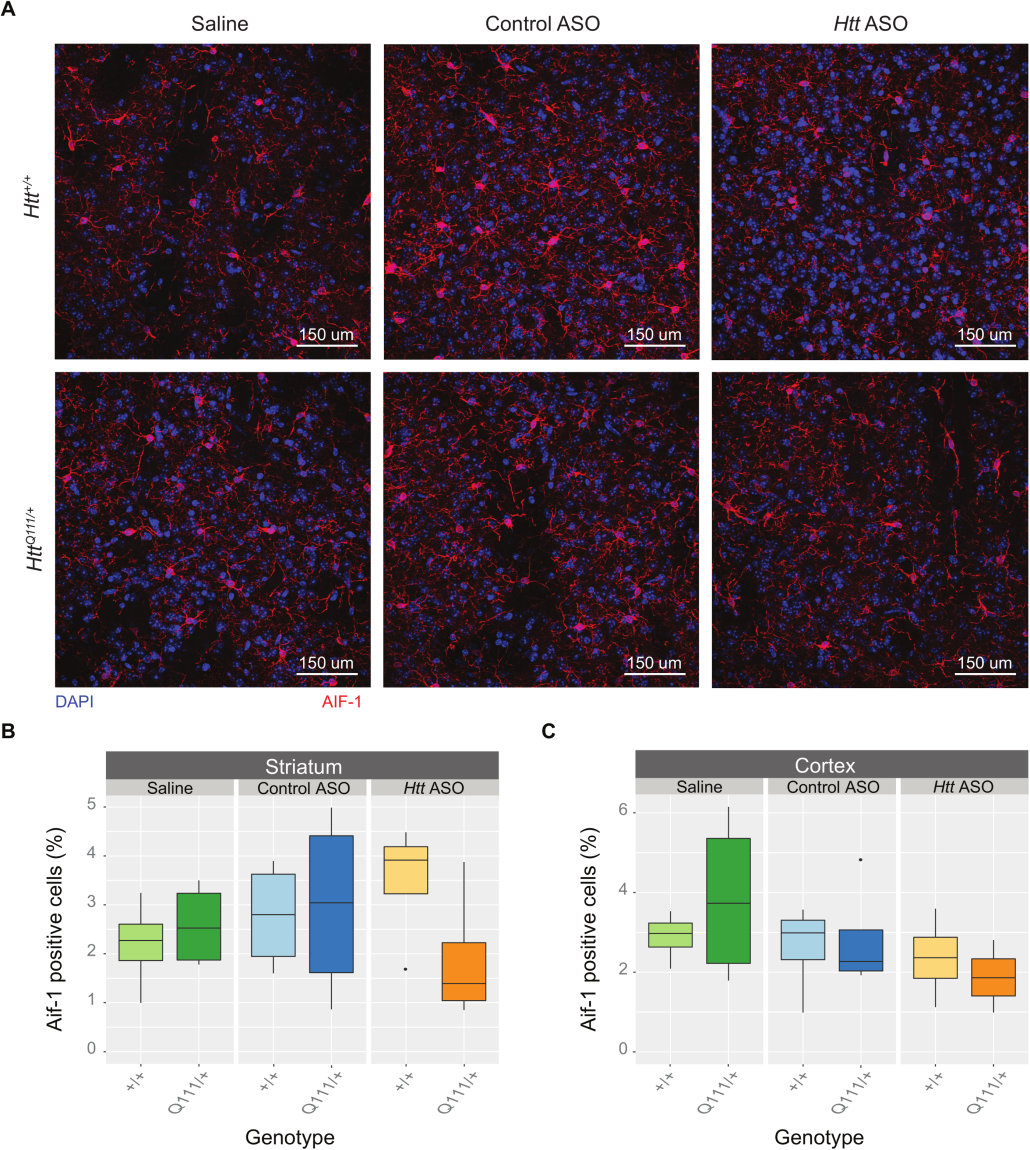
Peripheral *Htt* ASO treatment does not alter corticostriatal microglia density. (A) Representative images of AIF-1 (commonly referred to as IBA1) staining taken of the dorsolateral striatum highlight microglia (red) and nuclei (DAPI, blue) in every genotype and treatment condition. No differences were observed in the microglial counts between treatments or genotypes in the dorsolateral striatum (B) or deep cortical layers (C).

Enhanced microglial activation and proliferation is observed in several HD mouse models [29] as well as HD patients [30,31] and presymptomatic HD mutation carriers [32]. Although we do not detect increased microglial density in the dorsolateral striatum of 12-month *Htt*^Q111/+^ mice [16], immune consequences of peripheral *Htt* ASO treatment could in turn alter microglial proliferation in the central nervous system (Fig 4). However, we observe no changes in microglial density in the dorsolateral striatum (effect of genotype: *F*_(1,18)_ = 0.42, *p* = 0.52; effect of treatment: *F*_(2,18)_ = 0.29, *p* = 0.75; and genotype by treatment interaction: *F*_(2,18)_ = 1.48, *p* = 0.25) or deep cortical layers (effect of genotype: *F*_(1,18)_ = 0.18, *p* = 0.67; effect of treatment: *F*_(2,18)_ = 1.95, *p* = 0.17; and genotype by treatment interaction: *F*_(2,18)_ = 0.65, *p* = 0.53) after prolonged *Htt* ASO treatment.

**Fig 5:**
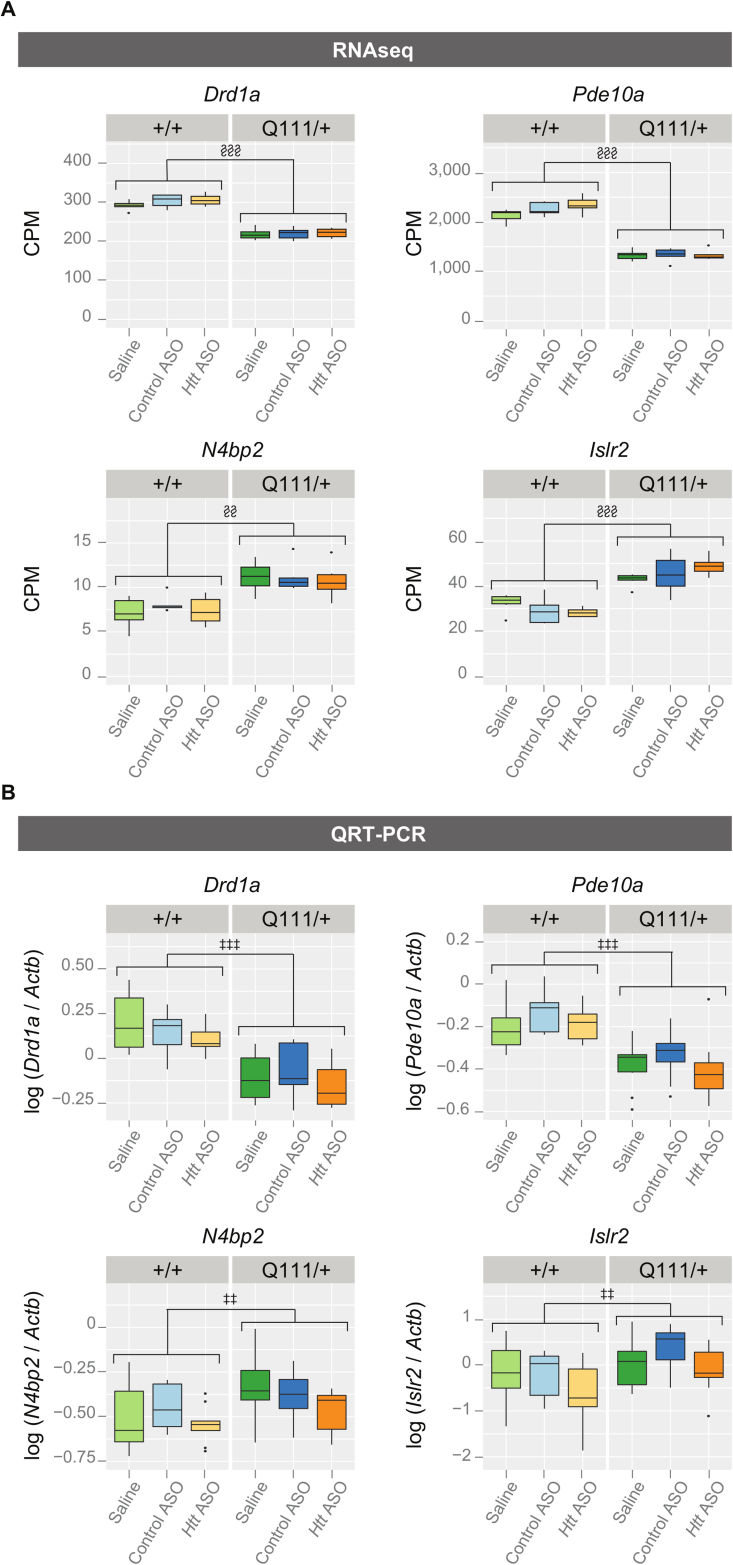
Peripheral *Htt* silencing does not rescue striatal transcriptional dysregulation in *Htt*^*Q111/+*^ mice. Using both RNAseq (A) and QRT-PCR (B), we assessed transcriptional markers of HD-like pathology in the striatum. Four transcripts, *Drd1a*, *Pde10a*, *N4bp2*, and *Islr2* were selected to illustrate the agreement between QRT-PCR and RNAseq results. We successfully replicated HD-relevant, steady state transcriptional changes previously characterized in *Htt*^*Q111/+*^ and wild-type mice, but *Htt* ASO treatment failed to rescue these phenotypes. ‡ p ≤ 0.05, ‡‡ p ≤ 0.01, ‡‡‡ p ≤ 0.001: by factorial ANOVA § FDR ≤ 0.05, §§ FDR ≤ 0.01, §§§ FDR ≤ 0.001 Abbreviations: counts per million (CPM).

Progressive, striatal-specific, transcriptional dysregulation is another feature of HD mouse models, including the *Htt*^*Q111/+*^ mouse [5,15,16]. We therefore examined striatal transcript alterations using both targeted (QRT-PCR) and untargeted (RNA Sequencing, RNASeq) methods. As expected, 10-month-old *Htt*^*Q111/+*^ mice have reduced steady state levels of key striatal synaptic and signaling genes, including *Drd1a* and *Pde10a* (Fig 5). In each case, transcript levels were reduced in the striatum of *Htt*^*Q111/+*^ mice relative to *Htt*^*+/+*^ mice using both RNASeq (*Drd1a*: *F*_(1,29)_ = 103.78, FDR = 1.66 x 10^−12^, *Pde10a*: *F*_(1,29)_ = 168.06, FDR = 5.94 x 10^−15^) and QRT-PCR detection methods (*Drd1a*: *F*_(1,53)_ = 67.97, *p* = 4.57 x 10^−11^, *Pde10a*: *F*_(1,53)_ = 44.47, *p* = 1.54 x 10^−8^). To ensure neither our QRT-PCR or RNAseq methods preferentially detected only downregulated genes, we quantified HD-relevant transcripts previously reported as upregulated in the striatum of *Htt*^*Q111/+*^ mice [16]. For instance, we successfully replicated increased steady state levels of *Islr2* and *N4bp2* in 10-month *Htt*^*Q111/+*^ mice (Fig 5) using both RNAseq (*Islr2*: *F*_(1,29)_ = 42.84, FDR = 3.45 x 10^−8^ and *N4bp2*: *F*_(1,29)_ = 10.96, FDR = 1.67 x 10^−3^) and QRT-PCR (*Islr2*: *F*_(1,52)_ = 10.65, *p* = 1.95 x 10^−3^ and *N4bp2*: *F*_(1,53)_ = 7.68, *p* = 7.68 x 10^−3^). Peripheral treatment with *Htt* ASO did not impact steady state expression levels of any of these transcripts, suggesting that striatal transcriptional dysregulation was not rescued by this treatment.

### Behavior

**Fig 6:**
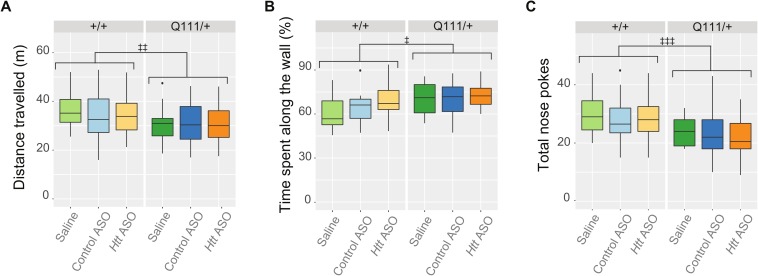
Hypoactivity and increased anxiety-like phenotypes are not improved by peripheral *Htt* silencing. During open field exploration, *Htt*^*Q111/+*^ mice travel less distance than *Htt*^*+/+*^ mice (A) and spend more time along the outer walls of the arena (B). This thigmotactic tendency combined with observations that *Htt*^*Q111/+*^ mice initiate fewer total investigations of both objects during the novel object location task (C) suggest that *Htt*^*Q111/+*^ mice exhibit anxiety-like phenotypes and are less motivated to explore. However, *Htt* ASO treatment failed to modulate these phenotypes. ‡ p ≤ 0.05, ‡‡ p ≤ 0.01, ‡‡‡ p ≤ 0.001: by factorial ANOVA.

One month prior to sacrifice, we investigated whether peripheral ASO treatment improved any HD-relevant behavioral changes in the *Htt*^*Q111/+*^ mouse. We first examined exploratory behavior in an open field task, testing mice during the first 8 hours of their dark cycle in an illuminated (475 lux) room. Consistent with previous investigations [26], exploratory activity in a 10 minute open field task was modestly reduced in *Htt*^*Q111/+*^ mice, compared to *Htt*^*+/+*^ mice (Fig 6A; 8.95% reduction, effect of genotype, *F*_(1,93)_ = 6.6, *p* = 0.01). There was no main effect of treatment (*F*_(2,93)_ = 0.3, *p* = 0.7) or genotype/treatment interaction (*F*_(2,93)_ = 0.3, *p* = 0.7), suggesting that peripheral *Htt* ASO treatment was not able to improve the mildly hypoactive phenotype observed in *Htt*^*Q111/+*^ mice. To determine if the reduction in exploratory activity observed in *Htt*^*Q111/+*^ mice may be caused by subtle motor deficit, we calculated the average velocity of each mouse and observe a mild reduction in *Htt*^*Q111/+*^ mice (4.9% reduction; *F*_(1,93)_ = 5.7, *p* = 0.02), but no effect of treatment (*F*_(2,93)_ = 0.5, *p* = 0.6) or genotype/treatment interaction (*F*_(2,93)_ = 0.2, *p* = 0.86). We also quantified thigmotaxis, the tendency to explore the outer walls of the open field arena compared to the center, as a proxy for anxiety levels [33]. We observed a modest increase in thigmotaxis in *Htt*^*Q111/+*^ mice (Fig 6B; 8.3% increase; genotype, *F*_(1,93)_ = 6.3, *p* = 0.01), but no effect of treatment (*F*_(2,93)_ = 1.9, *p* = 0.2) or genotype/treatment interaction (*F*_(2,93)_ = 0.6, *p* = 0.6). These results suggest that 9-month-old *Htt*^*Q111/+*^ mice are mildly hypoactive and potentially anxious, but that reducing peripheral HTT levels does not improve these phenotypes.

We next examined long term spatial memory in *Htt*^*Q111/+*^ mice using an object location task which exploits the tendency of rodents to preferentially explore an object that has been displaced to a novel location following a delay period, in this case 24 hours. In contrast to previous reports of impairments in small cohorts of 6-month old *Htt*^*Q111/+*^ mice [34], we find both *Htt*^*+/+*^ and *Htt*^*Q111/+*^ mice exhibit a preference for the object in a novel location even at 9 months of age (*Htt*^*+/+*^: *χ*^*2*^ = 50.4, *p* < .001; *Htt*^*Q111/+*^: *χ*^*2*^ = 23.1, *p* < .001). As a percent of total investigations, *Htt*^*Q111/+*^ and *Htt*^+/+^ mice explored objects in a novel location equally irrespective of genotype (*F*_(1,92)_ = 0.2, *p* = 0.7) or treatment (*F*_(2,92)_ = 0.8, *p* = 0.5), indicating that *Htt*^*Q111/+*^ mice have intact spatial long-term memory at 9 months of age, and that spatial long term memory is unaffected by *Htt* ASO treatment (S5 Fig). Interestingly, *Htt*^*Q111/+*^ mice conducted overall fewer investigations of either object location (19.1% reductions in total investigations, *F*_(1,92)_ = 14.15, *p* < 0.01, Fig 5C), suggesting a motivational deficit in *Htt*^*Q111/+*^ mice. However, *Htt* ASO treatment was unable to rescue this phenotype (*F*_(2,92)_ = 0.5, *p* = 0.6). These reductions suggest that previous reports of altered long-term memory could be confounded by neophobia or motivational changes in *Htt*^*Q111/+*^ mice at this age, rather than spatial memory deficits.

## Discussion

Given the ubiquitous expression of the mutant *Htt* allele, it is not surprising that peripheral phenotypes are widely observed in HD mutation carriers. We have suggested that peripheral dysfunction could potentially contribute directly to the progression of HD phenotypes in the CNS [9], a hypothesis we tested here by silencing *Htt* in peripheral organs. We find that effective silencing of hepatic and adipose *Htt* during a window in which the progression of central signs occurs rapidly [16] does not impact the progression disease signs in the CNS. These phenotypes include transcriptional dysregulation, accumulation of neuronal intranuclear inclusions, and HD-relevant behavioral changes.

Our experiments were designed to test whether direct reduction of mutant huntingtin in peripheral organs, rather than correction of downstream physiological perturbations, is generally beneficial for the CNS. Several previous studies in HD mouse models have examined the effect of peripherally-restricted interventions on the progression of some CNS-resident phenotypes. For example, imposition of a low-protein diet reduces circulating ammonia and citrulline levels and is associated with improved behavioral and CNS pathological signs of HD in both knock-in and transgenic mouse models [21]. Cross-genotype bone marrow transplants dampen peripheral immune activation in transgenic BACHD mice, and are associated with preservation of synapses and some behavioral benefits in that model [35]. Beyond HD, correction of splicing deficits and consequent increases in the level of SMN2 in mouse models of spinal muscular atrophy improves hepatic phenotypes and greatly extends survival compared to treatments limited to the CNS [36]. Similarly, in a mouse model of the polyglutamine expansion disease spinal-bulbar muscular atrophy, muscle expression of mutant androgen receptor was found to be required for disease-relevant phenotypes, excepting CNS protein aggregation [37]. Taken together with our results, it appears that the degree of cell autonomy for neurodegenerative disease phenotypes varies across both specific phenotypes and specific diseases, and is therefore worthy of careful study in each case.

We investigated behavioral, as well as pathological, signs of HD in the B6.*Htt*^*Q111/+*^ mouse model. In addition to modest hypoactivity and thigmotaxis observed in an open field task, we investigated long-term explicit spatial memory using an object location task (Fig 6). The *Htt*^*Q111/+*^ model has previously been reported to show deficits in long-term (24 hour) but not short-term (15 minute) object recognition memory. In the present study, we do not observe any deficit in long-term object location memory in *Htt*^*Q111/+*^ mice (S5 Fig), however we do observe an unexpected reduction in the total number of object investigations in 9-month-old *Htt*^*Q111/+*^ mice compared to *Htt*^*+/+*^ mice (Fig 6C). On average, *Htt*^*Q111/+*^ mice investigated objects in their environment 19% fewer times than *Htt*^*+/+*^ mice. Statistically, this is the most robust behavioral finding we have observed in *Htt*^*Q111/+*^ at this age. A range of behavioral studies suggest that motivational changes reminiscent of apathy are early phenotypes of knock-in models of HD [38] [39]. Prospective longitudinal study of HD mutation carriers reveals that apathy is unique among psychiatric symptoms in that it increases in severity across all stages of the disease, as well as being highly correlated with cognitive and motor impairment [40]. These findings suggest that our observations of reduced object investigations in *Htt*^*Q111/+*^ mice could point to early motivational changes, a hypothesis we are continuing to investigate with more targeted behavioral tasks.

Our study reveals that peripheral huntingtin silencing, or treatment with this specific ASO, results in modest reductions in body weight by 10 months of age (Fig 2). We have here limited our investigation to the impact of peripheral huntingtin silencing on CNS-resident signs of disease, but new evidence suggests that complete silencing of huntingtin in adult animals leads to peripheral phenotypes, including unexpected fatal pancreatitis [41]. Conversely, overexpression of wild-type huntingtin leads to robust increases in both body weight and the size of a number of organs including heart, liver, kidneys and spleen, suggesting huntingtin levels in peripheral tissues may have important physiological impacts worthy of continued investigation [42].

Our results demonstrate the reproducibility of previously described HD phenotypes in the *Htt*^Q111/+^ mouse [16], as well as the utility of this model in appropriately powered preclinical trials. Specifically, we aimed to test the link between peripheral organ pathology and central HD phenotypes. Although we achieved robust peripheral silencing of the HTT protein (approximately 67%) in the liver and adipose tissues, this intervention failed to rescue central signs of disease, including formation of neuronal intranuclear inclusions, transcriptional dysregulation, and behavioral phenotypes reminiscent of apathy.

## Methods

### Mice and genotyping

Female B6.*Htt*^Q111/+^ (RRID:IMSR_JAX:003456) and wild-type littermates were acquired from the Jackson Laboratories (Bar Harbor, ME). This strain is congenic on the C57BL/6J background and its creation been described previously [14]. CAG tract lengths of *Htt*^Q111/+^ mice ranged from 107–119 with an average of 114 (Table 1). Upon arrival at Western Washington University (WWU) at 3 weeks of age, mice were housed in a partially reversed light cycle, lights on from 12 am to 12 pm, with *ad libitum* access to food and water. Genotype and treatment were balanced across cages. After habituating for 5 weeks, treatment began at 2 months (61 ± 2 days) of age and lasted for 8 months. Two interim cohorts (n = 2 per arm, total N = 24) were established to verify HTT silencing prior to study completion at time points approximately one-third and two-thirds through the trial (at 11 and 22 weeks). All procedures were reviewed and approved by the animal care and use committee at WWU (protocol 14–006).

**Table 1:** CAG tract lengths of *Htt*^*Q111/+*^ mice in the efficacy cohort.

Treatment	Genotype	Measured CAG (SD)
*Htt* ASO	*Htt*^*Q111/+*^	114.5 (2.5)
Control ASO	*Htt*^*Q111/+*^	114.1 (2.7)
Saline	*Htt*^*Q111/+*^	113.3 (3.0)

For genotyping, genomic DNA was extracted from 3-mm tail biopsies taken at weaning. Presence or absence of the mutant allele was determined by polymerase chain reaction (PCR) using the primers CAG1 (5-’ATGAAGGCCTTCGAGTCCCTCAAGTCCTTC-3’) [43] and HU3 (5’-GGCGGCTGAGGAAGCTGAGGA-3’) [44], as previously described. PCR products were separated by gel electrophoresis in 2.2% agarose DNA cassettes (Lonza) and visualized using the FlashGel System.

### Antisense oligonucleotide administration

Ionis Pharmaceuticals supplied pan-*Htt*-targeting (Ionis 419637, ‘*Htt* ASO’) or off-target control (Ionis 141923, ‘control ASO’) ASOs, the latter with no sequence match in the mouse genome. Both ASOs were 20 nucleotide 5-10-5 2’-methoxyethyl gapmers with phosphorothioate backbones [17]. The sequences of *Htt* ASO and control ASO were 5’-CCTGCATCAGCTTTATTTGT-3’ and 5’-CCTTCCCTGAAGGTTCCTCC-3’ respectively (2’-methoxyethyl modified bases in the oligo ‘wings’ underlined). Mice were treated with 50 mpk *Htt* or control ASO via weekly IP injections, a dose and frequency selected based on initial dose-response and washout studies (S1 and S2 Figs). As an additional treatment control, a smaller cohort of mice were treated with 4 μL/g body mass of saline, a dose approximately equal in injection volume to that of *Htt* ASO and control ASO.

### Tissue harvesting

At 10 months of age, mice were sacrificed and tissues were harvested as described previously [16]. Briefly, a lethal injection of at least 250 mpk of sodium pentobarbital containing euthanasia solution was administered via IP injection, plasma was collected via cardiac puncture and centrifugation, and mice were transcardially perfused with phosphate buffered saline (PBS) to clear blood from organs. Liver, perigonadal white adipose tissue (WAT), and interscapular brown adipose tissue (BAT) were flash frozen for molecular analyses. Whole brain was removed and the hemispheres were separated along the longitudinal fissure. Using a mouse brain slicer matrix (Zivic), a consistent 3-mm corticostriatal block was cut from the left hemisphere and fixed overnight in 10% neutral buffered formalin (NBF) in preparation for paraffin embedding. To enable cutting of free-floating sections, the remaining posterior portion of the left hemisphere was also fixed overnight in NBF. Striatum, cortex, and cerebellum were dissected from the right hemisphere and flash frozen for molecular analyses.

### MSD assay

BioFocus, a Charles River company (Leiden, The Netherlands) quantified levels of both mutant and total HTT using previously described MSD assays [23]. Each assay utilized rabbit polyclonal pAB146 as the capture antibody, but differing sets of secondary antibodies in order to discriminate between polyglutamine expanded HTT and total HTT. Tissue lysates were divided and incubated in either the mouse monoclonal MW1 antibody (Developmental Studies Hybridoma Bank; Ab_528290) for mutant HTT assays or the rabbit polyclonal pAB137 antibody and the monoclonal mouse anti-HTT antibody (EMD Millipore: MAB2166; Ab_2123255) for total HTT assays. A goat anti-mouse SULFO TAG antibody was used to detect all secondary antibodies. HTT quantification was performed using flash frozen liver, WAT and BAT tissue from a subset of five *Htt*^+/+^ and *Htt*^Q111/+^ mice per arm of the efficacy trial. Likewise, hemi-striatum, liver, WAT and BAT from two mice per treatment and genotype condition in the interim silencing cohorts were used to assess total and mHTT knockdown mid-way through the efficacy trial.

### Behavior

Open field and object location testing occurred across 3 consecutive days in an open-field arena constructed of black acrylic walls and a white acrylic floor (44 x 44 x 44 cm^3^) with room lighting maintained at ~475 lux. Mice were moved to an experiment room, given 30 minutes to habituate, and tested between 1:00 and 8:00 PM. The arena and objects were cleaned between mice using 70% EtOH to minimize the effect of olfactory cues on exploratory behavior. On days 1 and 2, mice were habituated to the open-field arena in the absence of any objects by exploring freely for 10 minutes before being returned to their home cage. Activity was tracked using an overhead camera, and the exploratory behavior recorded during day 1 was analyzed using Noldus Ethovision XT 8 [45].

The object location task was conducted on day 3, as described [46]. Briefly, mice were placed into the open-field arena in the presence of two identical objects (Erlenmeyer flasks, terra cotta pots, Nalgene bottles filled with blue food coloring, and Nalgene bottles covered with textured coozies) located in the NW and NE corners of the arena. Objects were counterbalanced between animals to ensure equal representation between experimental conditions. Large intra-maze cues (a square, a triangle, an equal sign, and a plus) were taped to the N, S, E, and W walls respectively to provide spatial cues. Mice were placed into the arena facing N toward the objects and were permitted to explore for a single 10 minute acquisition period before being returned to their home cage. Following a 24-hour delay, one object was displaced to the diametrically opposite corner of the arena (i.e. if the NW object was displaced, it was moved to the SW corner, if the NE object was displaced, it was moved to the SE corner). Mice were placed into the arena facing N and permitted to explore for a single 5 minute probe session. The number of explorations toward each object, defined as orienting the head toward the object within 2 cm proximity or interacting directly with the object, was recorded by an experimenter blind to genotype and treatment.

### Transcriptional profiling

Total RNA was extracted from the hemistriatum as described previously [16] for both RNA sequencing (RNAseq) and quantitative reverse-transcription polymerase chain reaction (QRT-PCR). RNAseq was conducted at EA | Q^*2*^ Solutions with cDNA libraries prepared using the TruSeq Stranded mRNA sample preparation kit (Illumina # RS-122-2103) for 2x50bp PE sequencing. Quality control, as well as gene and isoform quantification, were performed with an EA | Q^*2*^ Solutions developed analysis pipeline, mRNA v7. Bowtie version 0.12.9 was used to align reads to the mouse transcriptome MGSCv37, followed by quantification with RSEM version 1.1.18. RNAseq data are publicly available via the Gene Expression Omnibus (GEO) accession number GSE97101.

For QRT-PCR, messenger RNA was reverse transcribed using the Superscript III First Strand Synthesis System (Life Technologies) according to the manufacturer’s protocol. QRT-PCR was conducted and analyzed as described [16] using the following taqman probes purchased from Life Technologies: Drd1a: Mm02620146\_s1, N4bp2: Mm01208882\_m1, Islr2: Mm00623260\_s1, Pde10a: Mm00449329\_m1, and β-actin: Mm02619580_g1. All transcripts were normalized to β-actin.

### Immunohistochemistry

For p62 staining, fixed corticostriatal blocks were paraffin embedded, cut into 5-μm sections, and mounted on glass slides for immunohistochemistry at Histology Consultation Services (Everson, WA). Deparaffinization, heat mediated antigen retrieval, and antibody staining were conducted as described previously [16]. For IBA-1 staining, posterior corticostriatal blocks were mounted in OCT compound (Tissue-Tek), cut into 40-μm free-floating sections, and stained as described [16]. All sections were imaged on an IX-81 laser-scanning confocal microscope. For each stain, acquisition settings were selected so that no primary controls emitted no fluorescent signal, and these settings were kept constant throughout imaging. ImageJ generated maximum z-projections using an equal number of sections for all images. To quantify p62-positive aggregates, experimenters blind to genotype and treatment selected a NeuN fluorescent threshold, which was used to create a NeuN mask and quantify p62-positive aggregates within each neuronal soma. Aggregates were automatically counted using ImageJ if they met experimenter-defined criteria for minimum fluorescence and size. Microglia cell bodies were automatically counted using ImageJ if they met experimenter-defined criteria and overlapped with an experimenter-defined DAPI mask.

### Statistical analyses

Data were analyzed using R version 3.3.1 [47]. Data presented in Figs 1, 2, 3B, 4, S3 and S5 were tested for normality (Shapiro-Wilk test) and homoscedasticity (Levene’s test). All data met or approximated parametric assumptions within groups and were fit with linear models analyzed by ANOVA. Data are presented as boxplots created with ggplot2 [48]—horizontal lines indicate 25th, 50th and 75th percentile, while the vertical whiskers indicate the range of data. Data falling outside 1.5 times the interquartile range are graphed as isolated points, but were not excluded from statistical analysis. RNASeq data were analyzed using the limma [49] package of Bioconductor [50] after normalizing with the voom function.

Weekly body weights were considered spurious and excluded if the *LNP* was greater than 130 for two or more consecutive weeks with *LNP* defined as

$$LNP=|100\;x\frac{lnW{}_{T}-lnW_{\left(T-1\right)}}{lnT-\ln\left(T-1\right)}|$$

where *W*_*T*_ is the weight at time *T* and *W*_(*T*−1)_ is the weight at time *T*−1. Using this criteria, less than 1% of observations were excluded. Body weight data were analyzed using a minP-based parametric bootstrap multiple comparison procedure [24,25]. The minP procedure provides adjusted *p*-values that correctly account for the family-wise error rate as the error control criterion based on the raw *p*-values calculated from Welch’s *t*-test. As the *p*-value adjustment requires knowledge of the underlying distribution of the minimum of *p*-values under the null hypothesis of no weight difference, we approximated the distribution using 10,000 parametric bootstrap resamples from the mean-zero multivariate normal distribution with the estimated covariance matrix [25]. Because the raw *p*-values are used as the test statistics in the minP procedure, we report the minimum of the raw *p*-values (*P*_min_) as the test statistic for the hypothesis of interest, and the adjusted *p*-value as the corresponding *p*-value.

## Supporting information

S1 Fig Treatment with 50 mpk or greater of *Htt* ASO effectively suppresses HTT in the liver. In order to determine an appropriate dose of *Htt* ASO, we conducted a preliminary dose response study and quantified HTT levels in the liver (our primary tissue of interest) via western blotting. Due to gel constraints, all doses could not be included on a single gel, therefore 17.9 and 35.8 mpk are presented in (A) while 50 and 100 mpk are presented in (B). Faint HTT bands remain in liver protein extracted from mice treated with 35.8 mpk per week (A), while treatment with 50 mpk per week or greater produced seemingly complete knockdown of HTT (B). Accordingly, 50 mpk was selected as the *Htt* ASO dose for the efficacy study.
Abbreviations: positive loading control (LC), huntingtin protein (HTT), β-Actin (β-Act).
(PDF)

S2 Fig *Htt* ASO mediated suppression lasts for 14 days after cessation of treatment in the liver. To roughly characterize the duration of action of our chosen ASO, we performed three, weekly IP injections of *Htt* ASO or off target ASO and measured liver HTT levels every other day for 24 days. Due to gel constraints, samples could not be loaded on a single gel, therefore days 2–12 are shown in (A) and days 14–24 are shown in (B). Based on these observations, we concluded weekly IP injections of *Htt* ASO were sufficient to ensure no recovery of HTT levels between treatments.
Abbreviations: positive loading control (LC), HTT: huntingtin protein (HTT), β-Act: β-Actin (β-Act).
(PDF)

S3 Fig ASO-mediated HTT suppression confirmed at intermediate timepoints throughout the efficacy trial, suggesting continuous HTT knockdown. Both total and mHTT levels were quantified via MSD assay in tissues harvested from two interim silencing cohorts at 4.5- and 7-months of age. As expected, the extent and pattern of HTT suppression was consistent with that seen in the efficacy trial, suggesting lapses in HTT knockdown during the efficacy trial are unlikely. Not surprisingly, mHTT levels were observed to be significantly higher in *Htt*^Q111/+^ mice than *Htt*^+/+^ mice across tissues. HTT levels following *Htt* ASO treatment were significantly reduced compared to saline or control treatment.
* p ≤ 0.05, ** p ≤ 0.01, *** p ≤ 0.001: by Tukey’s HSD pairwise comparisons Abbreviations: white adipose tissue (WAT), brown adipose tissue (BAT).
(PDF)

S4 Fig Qualitative assessment of peripheral *Htt* ASO distribution reveals efficient targeting of liver, spleen, and kidney. After 1 month of treatment with 50 mpk *Htt* ASO per week, mice were sacrificed and *Htt* ASO uptake was evaluated using an antibody reactive to the ASO backbone. Out of seven peripheral tissues, Htt ASO uptake was most pronounced in the liver, kidney and spleen, with modest uptake evident in the perigonadal white adipose tissue, gastrocnemius, interscapular brown adipose tissue and heart.
(PDF)

S5 Fig Regardless of treatment, *Htt^Q111/+^* and *Htt^+/+^* mice prefer objects in novel locations over familiar locations. In every genotype and treatment condition, mice explored objects located in the novel location over 50% of the time, demonstrating that spatial long term memory is neither impaired in *Htt*^*Q111/+*^ mice or affected by *Htt* ASO treatment.
(PDF)

S1 File Methods and results of ASO-dose response and wash out studies. (PDF)
