# Supplementary figures and images for: Peripheral huntingtin silencing does not ameliorate central signs of disease in the B6.HttQ111/+ mouse model of Huntington’s disease
Source: PLoS One. 2017 Apr 28;12(4):e0175968. doi: 10.1371/journal.pone.0175968 (PMC5409169; doi:10.1371/journal.pone.0175968)

**A**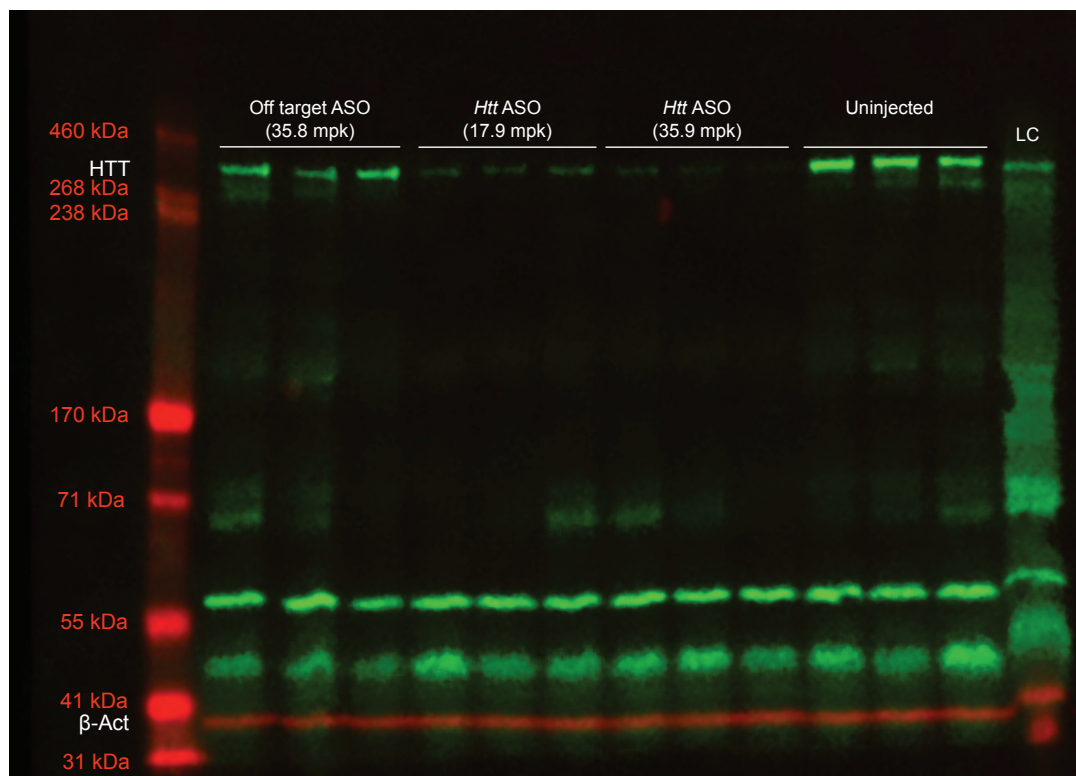**B**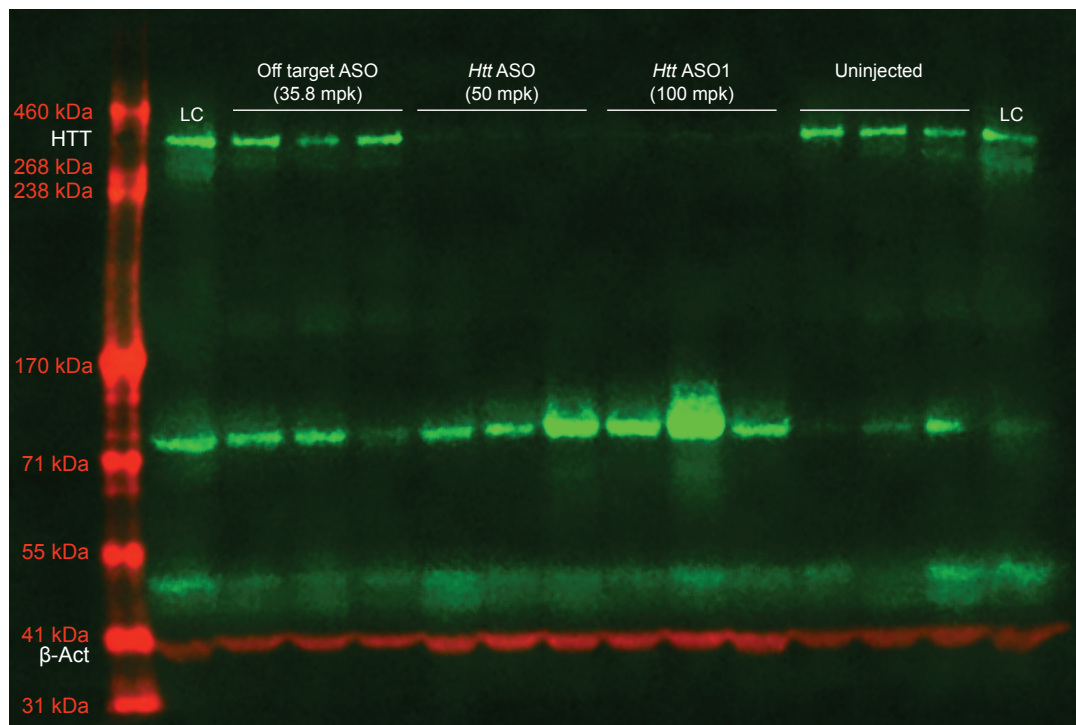

Supplement: S1 Fig — In order to determine an appropriate dose of Htt ASO, we conducted a preliminary dose response study and quantified HTT levels in the liver (our primary tissue of interest) via western blotting. Due to gel constraints, all doses could not be included on a single gel, therefore 17.9 and 35.8 mpk are presented in (A) while 50 and 100 mpk are presented in (B). Faint HTT bands remain in liver protein extracted from mice treated with 35.8 mpk per week (A), while treatment with 50 mpk per week or greater produced seemingly complete knockdown of HTT (B). Accordingly, 50 mpk was selected as the Htt ASO dose for the efficacy study. Abbreviations: positive loading control (LC), huntingtin protein (HTT), β-Actin (β-Act). (PDF) [file pone.0175968.s001.pdf]

HTT (fmol / mg total protein)

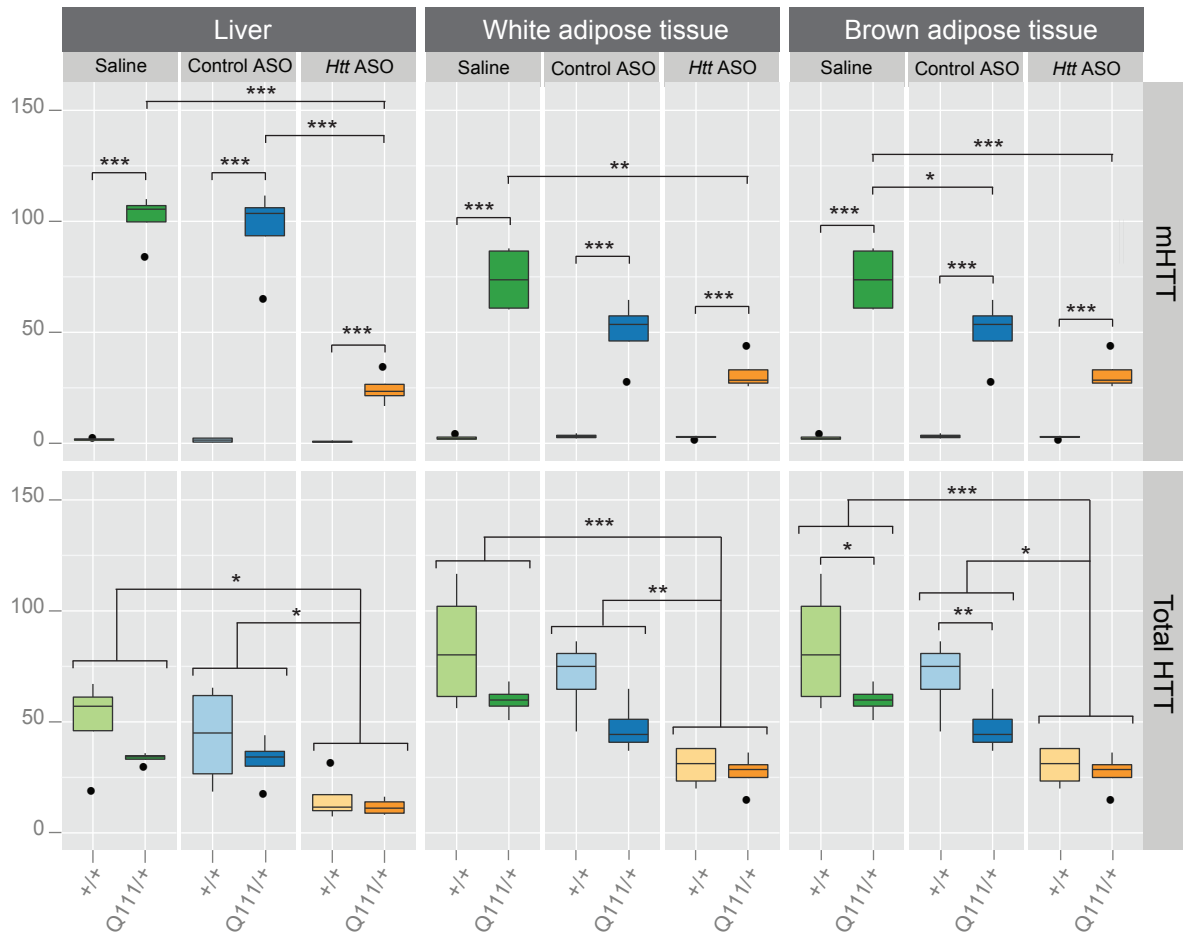

Supplement: S3 Fig — Both total and mHTT levels were quantified via MSD assay in tissues harvested from two interim silencing cohorts at 4.5- and 7-months of age. As expected, the extent and pattern of HTT suppression was consistent with that seen in the efficacy trial, suggesting lapses in HTT knockdown during the efficacy trial are unlikely. Not surprisingly, mHTT levels were observed to be significantly higher in HttQ111/+ mice than Htt+/+ mice across tissues. HTT levels following Htt ASO treatment were significantly reduced compared to saline or control treatment. * p ≤ 0.05, ** p ≤ 0.01, *** p ≤ 0.001: by Tukey’s HSD pairwise comparisons Abbreviations: white adipose tissue (WAT), brown adipose tissue (BAT). (PDF) [file pone.0175968.s003.pdf]

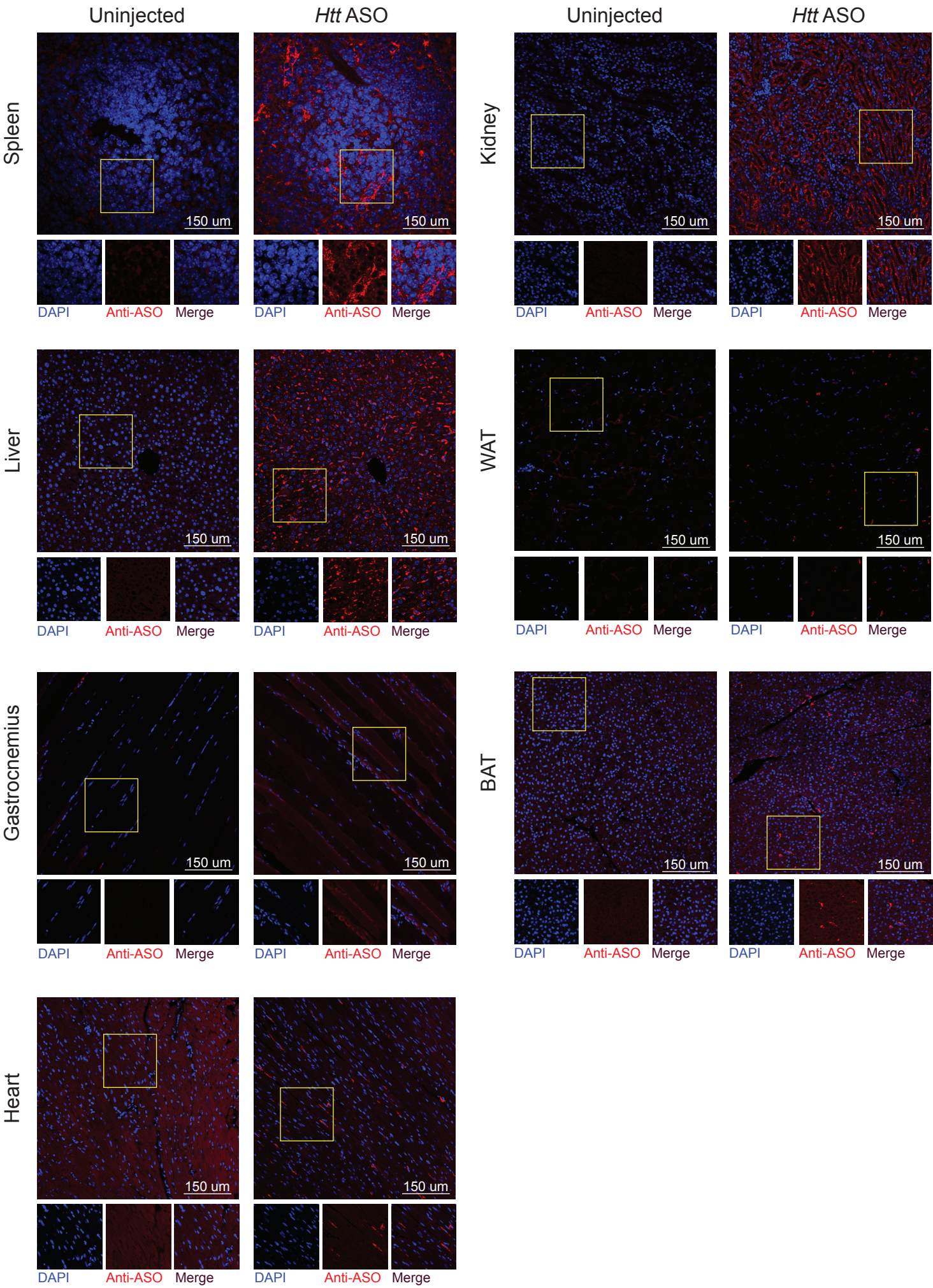

Supplement: S4 Fig — After 1 month of treatment with 50 mpk Htt ASO per week, mice were sacrificed and Htt ASO uptake was evaluated using an antibody reactive to the ASO backbone. Out of seven peripheral tissues, Htt ASO uptake was most pronounced in the liver, kidney and spleen, with modest uptake evident in the perigonadal white adipose tissue, gastrocnemius, interscapular brown adipose tissue and heart. (PDF) [file pone.0175968.s004.pdf]

Novel nose pokes (%)

+/+

Q111/+

Saline

Control ASO

Htt ASO

Saline

Control ASO

Htt ASO

100

75

50

25

0

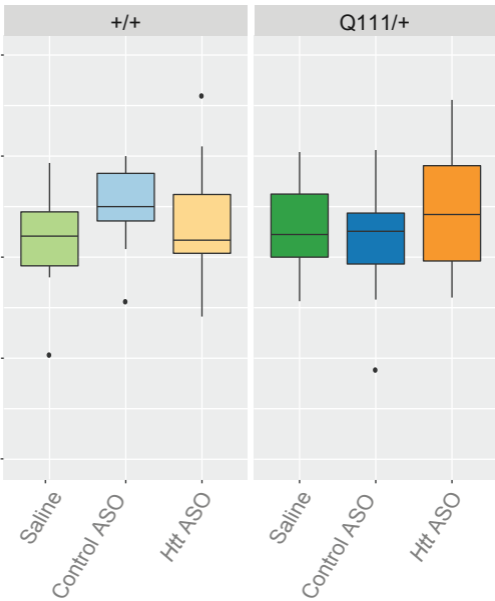

Supplement: S5 Fig — In every genotype and treatment condition, mice explored objects located in the novel location over 50% of the time, demonstrating that spatial long term memory is neither impaired in HttQ111/+ mice or affected by Htt ASO treatment. (PDF) [file pone.0175968.s005.pdf]
