# Supplementary material for: Peripheral huntingtin silencing does not ameliorate central signs of disease in the B6.HttQ111/+ mouse model of Huntington’s disease
Source: PLoS One. 2017 Apr 28;12(4):e0175968. doi: 10.1371/journal.pone.0175968 (PMC5409169; doi:10.1371/journal.pone.0175968)
Supplement: S2 Fig — To roughly characterize the duration of action of our chosen ASO, we performed three, weekly IP injections of Htt ASO or off target ASO and measured liver HTT levels every other day for 24 days. Due to gel constraints, samples could not be loaded on a single gel, therefore days 2–12 are shown in (A) and days 14–24 are shown in (B). Based on these observations, we concluded weekly IP injections of Htt ASO were sufficient to ensure no recovery of HTT levels between treatments. Abbreviations: positive loading control (LC), HTT: huntingtin protein (HTT), β-Act: β-Actin (β-Act). (PDF) [file pone.0175968.s002.pdf]

A

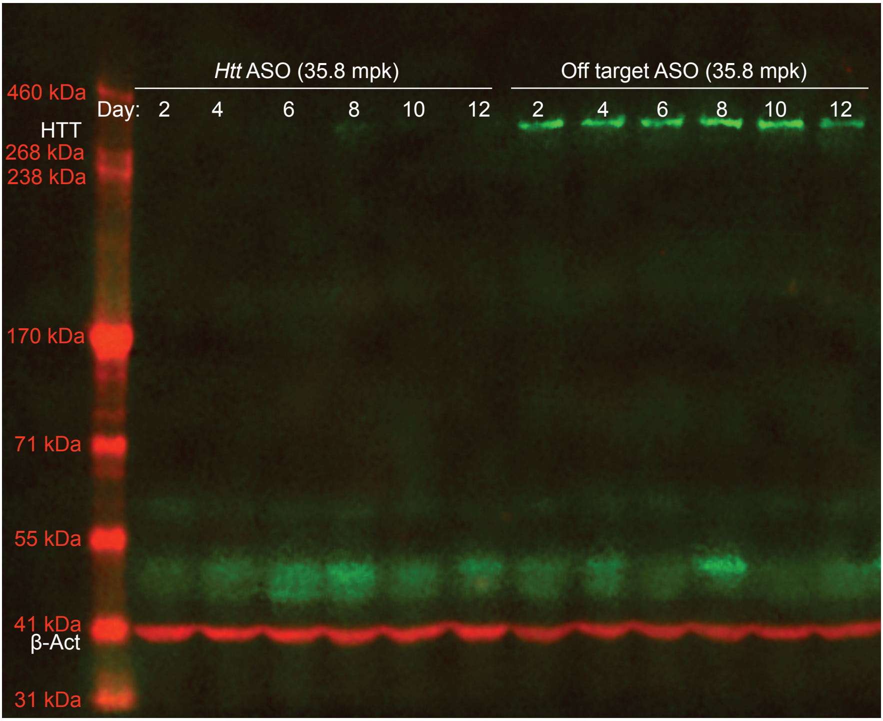

Htt ASO (35.8 mpk)
Off target ASO (35.8 mpk)
Day: 2 4 6 8 10 12 2 4 6 8 10 12
460 kDa
HTT
268 kDa
238 kDa
170 kDa
71 kDa
55 kDa
41 kDa
β-Act
31 kDa

B

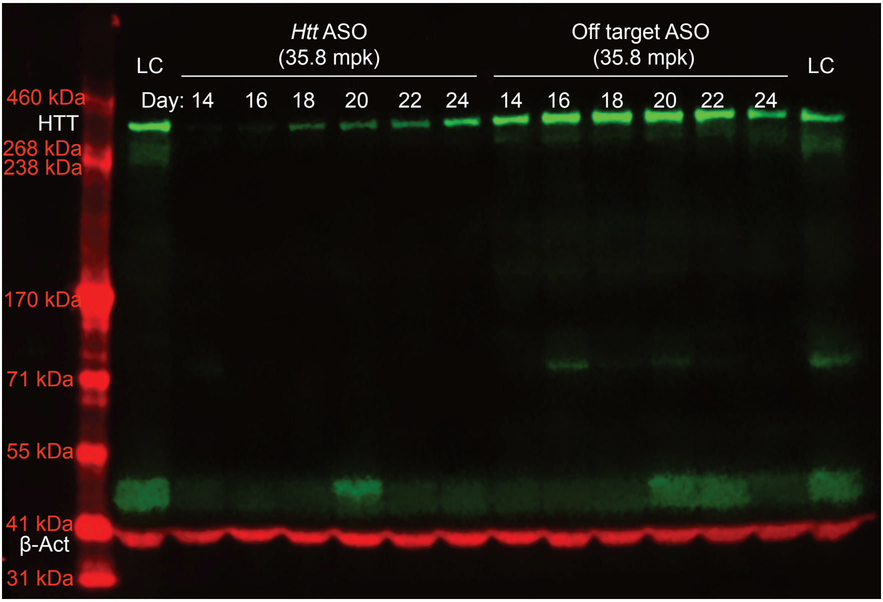

LC
Htt ASO (35.8 mpk)
Off target ASO (35.8 mpk)
LC
Day: 14 16 18 20 22 24 14 16 18 20 22 24
460 kDa
HTT
268 kDa
238 kDa
170 kDa
71 kDa
55 kDa
41 kDa
β-Act
31 kDa
