# Supplementary material for: Peripheral huntingtin silencing does not ameliorate central signs of disease in the B6.HttQ111/+ mouse model of Huntington’s disease
Source: PLoS One. 2017 Apr 28;12(4):e0175968. doi: 10.1371/journal.pone.0175968 (PMC5409169; doi:10.1371/journal.pone.0175968)
Supplement: S1 File — (PDF) [file pone.0175968.s006.pdf]

Peripheral huntingtin silencing does not ameliorate central signs of disease in the B6.*Htt*$^{Q111/+}$ mouse model of Huntington's disease

Sydney R. Coffey [1], Robert M. Bragg [1], Shawn Minnig [1], Seth A. Ament [2,3], Jeffrey P. Cantle [1], Anne Glickenhaus [1], Daniel Shelnut [4], José M. Carrillo [1], Dominic D. Shuttleworth [1], Julie-Anne Rodier [5], Kimihiro Noguchi [4], C. Frank Bennett [6], Nathan D. Price [2], Holly B. Kordasiewicz [6] and Jeffrey B. Carroll [1,*]

[1] Behavioral Neuroscience Program, Psychology Department, Western Washington University, Bellingham, WA, USA

[2] Institute for Genome Sciences and Department of Psychiatry, University of Maryland School of Medicine, Baltimore, MD, USA

[3] Institute for Systems Biology, Seattle, WA, USA

[4] Department of Mathematics, Western Washington University, Bellingham, WA, USA

[5] INSERM U1216, Grenoble Institute of Neuroscience, Grenoble, France.
Université Grenoble Alpes, Grenoble, France.

[6] Ionis Pharmaceuticals, Carlsbad, CA, USA

* Corresponding author

Jeff.Carroll@wwu.edu (J.B.C.)

Results

Dose-response and washout studies

We piloted four different doses of *Htt* ASO and qualitatively assessed HTT knockdown by western blotting. At the two highest doses, no HTT could be detected, despite robust HTT signal in protein lysates extracted from off target ASO and uninjected mice (Figure in S1 Figure). Therefore we selected 50 mg per kg body mass (mpk) per week as the dose for the efficacy study; this was the minimal dose at which complete HTT knockdown was observed. β-Actin was used as a loading control and was successfully detected in all samples regardless of treatment.

To determine how frequently *Htt* ASO must be administered in order to maintain constant HTT knockdown, we conducted a washout study. Wild type mice were given weekly injections of *Htt* ASO or off target ASO for three weeks and sacrificed every other day for 24 days. During this time, we observed complete HTT silencing for 14 days after cessation of treatment (Figure in S2 Figure), and even 24 days after the final injection, HTT did not recover to physiological levels. For the efficacy trial, we chose to conservatively administer *Htt* ASO via weekly injections.

HTT knockdown in interim silencing cohorts

At two points midway through the efficacy trial, we confirmed HTT knockdown in three peripheral tissues of interest: liver, perigonadal white adipose tissue, and interscapular brown adipose tissue. HTT levels were quantified via mesoscale discovery (MSD) assays, as described in the primary manuscript. No differences in mutant or total HTT suppression patterns were observed between timepoints, therefore data were grouped for statistical analysis. *Htt* ASO treatment had a significant effect on total HTT levels (Figure in S3 Figure) in all three tissues (effect of treatment in the liver: $F_{(2, 18)}$ = 9.8, p = 1.8 x 10^{-3}, white adipose tissue: $F_{(2, 18)}$ = 15.7, p = 1.2 x 10^{-4}, and brown adipose tissue: $F_{(2, 18)}$ = 11.3, p = 6.5 x 10^{-4}). As expected, Tukey's HSD *post hoc* comparisons revealed *Htt* ASO treatment suppressed HTT compared to both control ASO (liver: p = 8.8 x 10^{-3}, white adipose tissue: p = 3.1 x 10^{-3}, and brown adipose tissue: p = 2.2 x 10^{-3}) and saline treatment (liver: p = 2.6 x 10^{-3}, white adipose tissue: p = 1 x 10^{-4}, and brown adipose tissue: p = 1.4 x 10^{-3}). Similarly, mHTT was significantly reduced by *Htt* ASO treatment in all tissues quantified (effect of treatment in the liver: $F_{(2, 18)}$ = 36.8, p = 4.4 x 10^{-7}, white adipose tissue: $F_{(2, 18)}$ = 9.6, p = 1.4 x 10^{-3}, and brown adipose tissue: $F_{(2, 18)}$ = 5.6, p = 0.01). Tukey's HSD *post hoc* tests verified reduction of mHTT following *Htt* ASO treatment compared to saline treatment (liver: p = 1.2 x 10^{-6}, white adipose tissue: p = 1.0 x 10^{-3}, and brown adipose tissue: p = 0.01). Interestingly, no difference in mHTT levels was observed by Tukey's HSD *post hoc* comparisons between *Htt* ASO and control ASO treatment in either of the adipose tissues (white adipose tissue: p = 0.16, and brown adipose tissue: p = 0.11), despite *Htt* ASO treatment reducing mHTT levels relative to control treatment in the liver (p = 3.5 x 10^{-6}).

Methods

Mice

In order to determine an appropriate ASO dose and frequency of administration, we conducted dose-response (n = 5 per arm, total N = 30) and washout studies (n = 1 per arm, total N = 24) in male, wild-type C57BL/6J mice (stock no. 000664) acquired from the Jackson Laboratories (Bar Harbor, ME). Mice were housed under standard vivarium conditions with *ad libitum* access to food and water. After shipment, mice habituated to local vivarium conditions for at least two weeks prior to every study. For both the washout and dose-response studies, treatment began at three months of age and continued for three weeks. For the dose-response study, mice were sacrificed two days after the final injection as described in the primary manuscript. For the washout study, mice were sacrificed every other day after cessation of treatment.

ASO uptake in peripheral tissues was qualitatively assessed in a small cohort made up of female, C57BL/6J mice bred locally (n = 2 per arm, total N = 4). Mice in the tissue distribution cohort were maintained with *ad libitum* access to food and water. Treatment began at 4 months (121 ± 1 days) of age and continued for four weeks. At 5 months (147 ± 4 days) of age, or two days after cessation of treatment, mice were sacrificed via intraperitoneal injection of at least 250 mpk sodium pentobarbital and transcardially perfused with phosphate buffered saline (PBS). Liver, perigonadal white adipose tissue (WAT), interscapular brown adipose tissue (BAT), kidney, spleen, heart, and gastrocnemius were dissected and fixed overnight in 10% neutral buffered formalin.

All procedures were reviewed and approved by the animal care and use committee at Western Washington University (protocol 14-006).

Antisense Oligonucleotide Administration

In an initial dose-response study, we administered a pan-*Htt* targeted ASO (Ionis 419637, *Htt* ASO) via weekly IP injection at four different doses: 17.9, 35.8, 50, or 100 mpk. As a control, an off target ASO was administered weekly at 35.8 mpk. The off target ASO was selected as a control because it shares a similar chemical structure with *Htt* ASO, both of which are 20 nucleotide 5-10-5 2'-methoxyethyl gapmers. In addition, we compared both *Htt* ASO and off target ASO treated mice to uninjected mice. Based on this initial dose-response study, we selected a dose of 50 mpk per week for all subsequent studies.

To roughly characterize *Htt* ASO duration of action, we conducted a washout study in which mice were treated with *Htt* ASO or off target ASO at 35.8 mpk per week for three weeks. After cessation of treatment, mice were sacrificed every other day for 24 days and HTT levels quantified by western blot.

For the tissue distribution study, mice received weekly injections of *Htt* ASO at 50 mpk for four weeks.

Immunoblotting

Tissue (half of dissected brown adipose tissue and 35 - 55 mg of liver tissue) was transferred to BeadBug tubes prefilled with 1.5-mm zirconium oxide beads (Benchmark) and homogenized for 2 minutes at 4,000 rpm using the BeadBug Microtube

Homogenizer (Benchmark). Protein was extracted using 450 µL of RIPA buffer (150 mM NaCl, 25 mM Tris-HCl, 1% NP-40, 1% sodium deoxycholate, 0.1% SDS) containing protease and phosphatase inhibitors (Thermo Fisher Scientific). Concentration of protein lysates was determined by Pierce BCA assay (Thermo Fisher Scientific) according to the manufacturer's protocol.

Total HTT levels were quantified via western blotting. Equal amounts of protein were loaded into 3-8% Tris-Acetate Mini Gels (Thermo Fisher Scientific) and separated electrophoretically by molecular weight. Protein was transferred at 4℃ for 17 – 20 hours to a PVDF membrane.

Western blot assay membranes were blocked for 1 hour at room temperature using Odyssey Blocking Buffer (LiCor) before incubating for 1 hour at room temperature or overnight at 4℃ in primary antibodies. Monoclonal primary antibodies were diluted 1:1,000 in Odyssey blocking buffer plus 0.2% tween and consisted of mouse anti-HTT (EMD Millipore: MAB2166; Ab_2123255) and rabbit anti-β-Actin (Cell Signalling: mAB#8457, Ab_10950489). Membranes were rinsed three times in PBS plus 0.05% tween (PBST), then incubated in secondary antibodies for 1 hour at room temperature. Polyclonal secondary antibodies were IRDye 800CW conjugated goat anti-mouse IgG (LiCor: 926-32210; Ab_621842) and IRDye 680RD conjugated goat anti-rabbit IgG (LiCor: 926-68071; Ab_10956166) diluted 1:15,000 in Odyssey Blocking Buffer plus 0.2% tween and 0.01% SDS. After rinsing three times in PBST, membranes were washed in PBS before imaging for 2 minutes each at 700 and 800 nm.

Immunohistochemistry and Imaging

As part of the tissue distribution study, formalin-fixed liver, WAT, BAT, gastrocnemius, heart, spleen, and kidney tissues were paraffin embedded, cut into 5-µm sections, and mounted on glass slides at Histology Consultation Services (Everson, WA). Sections were deparaffinized and heat mediated antigen retrieval was conducted in Tris-EDTA buffer before staining overnight in anti-ASO primary antibody (1:1,000) at 4℃. Sections were stained in goat anti-rabbit secondary antibody conjugated to Alexa568 (1:1,000) for one hour at room temperature and mounted with DAPI Fluoromount-G (Southern Biotech). All confocal images were acquired with an IX-81 laser-scanning confocal microscope using Fluoview 1000 software (Olympus). Image processing and analysis was performed as described [1].

References

1. Bragg RM, Coffey SR, Weston RM, Ament SA, Cantle JP, Minnig S, et al. Motivational, proteostatic and transcriptional deficits precede synapse loss, gliosis and neurodegeneration in the B6.Htt(Q111/+) model of Huntington's disease. Sci Rep. 2017;7: 41570.
